# Dataset on maternal attitudes about child maltreatment in nine countries using a Q-sort methodology

**DOI:** 10.1016/j.dib.2020.105396

**Published:** 2020-03-10

**Authors:** Mi-lan Woudstra, Joost van Ginkel, Marjolein Branger, Rosanneke Emmen, Lenneke Alink, Faramarz Asanjarani, Rodrigo Carcamo, Celia Hsiao, Cindy Mels, Bilge Selcuk, Isabel Soares, Lamei Wang, Melis Yavuz, Judi Mesman

**Affiliations:** a Institute of Education and Child Studies, Leiden University, the Netherlands; b Institute of Psychology, Leiden University, the Netherlands; c Department of Counseling, Faculty of Education and Psychology, University of Isfahan, Iran; d University of Magallanes, Punta Arenas, Chile; e Department of Paediatrics, University of the Witwatersrand, Johannesburg, South Africa; f Department of Developmental and Educational Psychology, Catholic University of Uruguay, Montevideo, Uruguay; g Department of Psychology, Koc University, Istanbul, Turkey; h School of Psychology, University of Minho, Campus de Gualtar, Braga, Portugal; i College of Psychology and Sociology, Shenzhen University, China; j Department of Psychology, MEF University, Turkey

**Keywords:** Child maltreatment, Attitudes, Mothers, Culture, Q-sort

## Abstract

Analyses of the present data are reported in the article “Crossing Boundaries: A Pilot Study of Maternal Attitudes about Child Maltreatment in Nine Countries” [8]. Data were collected during home visits using the Maltreatment Q-Sort (MQS). A total of 466 mothers from nine different countries gave their opinion about child maltreatment by sorting 90 cards with parenting behaviors taken from the literature that reflect four types of child maltreatment, into 9 evenly distributed stacks (with 10 cards each) from least to most harmful for the child. This data article provides an overview of the content of the 90 items, which type of maltreatment they reflect, and the source of the items. The percentage of mothers labelling each of the MQS items as maltreatment is also presented. In addition, instructions are included about the administration of the MQS as well as data-entry and analyses of Q-sort data, accompanied by example datasets and syntaxes. This can serve as a manual for researchers interested in using Q-sort data.

**Specification table**

**Table:** 

Subject	Psychology
Specific subject area	Maternal Attitudes about Child Maltreatment
Type of data	Tables Figures
How data were acquired	Data were acquired during home visits, using the Maltreatment Q-sort and a survey covering family background (online or during the home visit).
Data format	Raw Analyzed Filtered (basic variables only)
Parameters for data collection	Participants were mothers with at least one child between 2 and 6 years old. Mothers could not participate when they had an ethnic minority status, a (target) child with a severe mental or physical disability, or were illiterate.
Description of data collection	Various methods (e.g., personal networks, snowball sampling, and social media) were used to recruit participants from nine countries. For data collection participants were visited at home. Mothers filled in a short survey about some socio-demographic variables (online before the home visit or during the home visit). In addition, to measure participants’ attitudes about child maltreatment the Maltreatment Q-Sort (MQS) was used. The MQS consists of a set of 90 items reflecting different types of child maltreatment which parents had to sort from least to most harmful to the child.
Data source location	Institution: • Institute of Education and Child Studies, Leiden University • Department of Counseling, Faculty of Education and Psychology, University of Isfahan • University of Magallanes • Department of Paediatrics, University of the Witwatersrand • Department of Developmental and Educational Psychology, Catholic University of Uruguay • Department of Psychology, Koc University + Department of Psychology, MEF University • School of Psychology, University of Minho • College of Psychology and Sociology, Shenzhen University City/Town/Region: • Western region of the Netherlands • Arak and Neishabour • Punta Arenas city • Greater Johannesburg Metropolitan Area • Montevideo • Istanbul and Izmir • Aveiro, Porto, and Braga • Shenzhen • Suburbs of Athens Country: • the Netherlands • Iran • Chile • South Africa • Uruguay • Turkey
	• Portugal • China • Greece
Data accessibility	With the article
Related research article	Author's name: Judi Mesman, Marjolein Branger, Mi-lan Woudstra, Rosanneke Emmen, Faramarz Asanjarani, Rodrigo Carcamo, Celia Hsiao, Cindy Mels, Bilge Selcuk, Isabel Soares, Joost van Ginkel, Lamei Wang, Melis Yavuz, Lenneke Alink Title: Crossing Boundaries: A Pilot Study of Maternal Attitudes about Child Maltreatment in Nine Countries Journal Child Abuse & Neglect DOI 10.1016/j.chiabu.2019.104257

## Value of the Data

• Definitions of and opinions about child maltreatment vary between countries. The data can be used to get more insight in differences and similarities in maternal attitudes about child maltreatment within and between countries.
• Researchers in the field of child maltreatment can benefit from these data, but also professionals working with families with different cultural backgrounds to enhance their understanding of attitudes mothers may have about child maltreatment.
• The data can be used to create new insights to design culturally sensitive interventions that target maternal attitudes about potentially harmful parenting behaviour.
• Because this data article includes the methodology of administering and analysing Q-sort data, it can serve as an example for researchers interested in using Q-sort data regardless of the specific topic.

## 1 Data

A number of datasets and syntaxes are provided. The first dataset [1] is an example of how Q-sort data should be entered. Two syntaxes [2,3] are needed to restructure the entered data into a ‘participants-as-variables’ format to be able to analyse Q-sort data. An example of the restructured data is provided [4]. In this data file each column represents one mother and each row represents one MQS card (1–90), each with scores from 1 to 9 to reflect the stack number on which the mother has placed the MQS cards. These data can be used to calculate the agreement between mothers, within and between counties, on how they sorted the 90 MQS cards. Again two syntaxes [5,6] are needed to calculate the agreement between the Q-sorts of the participants. The third dataset [7] includes an example of what a data file with the agreement between participants from different groups should look like. An explanation of how to use these datasets and syntaxes to analyze Q-sort data is provided in the sections ‘preparing data for analyses’ and ‘data analyses’.

In addition, two datasets with data presented in the paper of Mesman et al. [8] are available. These datasets contain data on attitudes about child maltreatment of 466 mothers from Chile (*n* = 49), China (*n* = 50), Greece (*n* = 45), Iran (*n* = 45), the Netherlands (*n* = 65), Portugal (*n* = 57), South Africa (*n* = 49), Turkey (*n* = 51), and Uruguay (*n* = 55). The first dataset [9] is a ‘participants-as-variables’ SPSS data file in which variables represent the mothers and cases represent the MQS cards with the associated stack number (1–9) on which the mothers placed each of the 90 MQS cards. The second dataset [10] is an ‘items-as-variables’ SPSS data file in which each row represents a participant and each column a variable. This dataset contains background variables of the participants, including the number of children, years of education, income, and age of the participants. For some countries there is also data available about from which of the 9 stacks onwards the participants think someone, themselves or a professional should intervene, and from which stack onwards they think the behaviors on the cards can be labelled as child maltreatment. For all mothers the dataset contains the stack number on which they placed each card and also the average stack number on which they placed the cards related to four subscales of child maltreatment (physical neglect, physical abuse, emotional neglect, and emotional abuse). Table 1 gives an overview of the item numbers with the associated content of the items, the MQS-scale to which the items belongs, and the source from which the items was taken. Table 2 shows the percentage of mothers (in the five countries with available data about threshold for defining maltreatment) who labelled the MQS items as maltreatment (see Mesman et al. [8] for a more detailed interpretation of the Table).

**Table 1:** Item Number, Items Content, Type, and Source of all 90 MQS Items.

Item #	Item content	Type^a^	Source
1	Is unable to offer the child a safe home.	PN	NPM^b^ 12.4
2	Gives the child so much food, that the child has an unhealthy weight.	filler	n.a.
3	Allows the child to meet with people who are drunk.	EN	NPM 16.1
4	Does not react to the child's emotions.	EN	NPM 17.5
5	Does not offer enough structure to the child.	EN	NPM 17.4
6	Does not intervene when the child is aggressive.	EN	NPM 16.2
7	Does not make the child feel important.	EN	CTQ^c^-EN/NPM 15.1
8	Is verbally aggressive towards the child.	EA	NPM 06.1
9	Belittles the child.	EA	NPM 06.1
10	Purposely destroys the child's favorite toys.	EA	NPM 07.1
11	Emotionally abuses the child.	EA	CTQ-EA/NPM all
12	Calls the child dumb or lazy.	EA	CTSPC^d^-PsA^e^/CTQ-EA/NPM 06.1
13	Threatens to spank or hit the child, but does not actually do it.	EA	CTSPC-PsA/06.3
14	Does not provide adequate care when the child is ill.	PN	NPM 10.1
15	Does not provide the child with a safe environment.	PN	NPM 10.4
16	Refuses to offer the child shelter.	PN	NPM 10.1
17	Is not able to make sure the child goes to a doctor or hospital when he/she needs it.	PN	CTSPC-N^f^/CTQ-PN/NPM 09.0
18	Uses a weapon to hit the child.	PA	NPM 04.3
19	Hits the child so hard that it leaves bruises.	PA	CTQ-PA/ NPM04.2/0.3
20	Threatens the child with a knife or gun.	PA	CTSPC-VSPA^g^/NPM 06.3
21	Kicks the child hard.	PA	CTSPC-SPA^h^/NPM 04.5
22	Slaps the child on the face or head or ears.	PA	CTSPC-SPA/NPM 04.2
23	Spanks the child on the bottom with bare hand.	PA	CTSPC-MPA^i^/NPM 04.2
24	Shouts, yells, or screams to another family member in front of the child.	EN	NPM 16.2
25	Is emotionally unavailable for the child.	EN	NPM 15.1
26	Uses illegal drugs in the presence of the child.	EN	NPM 17.6
27	Fails to find treatment that the child needs for an emotional or behavioral problem.	EN	NPM 17.2
28	Does not feel close to the child.	EN	CTQ-EN/NPM 15.1
29	Is so caught up in his/her own problems that he/she is not able to show or tell the child that he/she loves the child.	EN	CTSPC-N/NPM 15.1
30	Locks the child in a closet as a punishment.	EA	NPM 05.2
31	Humiliates the child in front of others.	EA	NPM 06.1
32	Threatens to kill the child.	EA	NPM 06.3
33	Says hurtful things to the child.	EA	CTQ-EA/NPM 06.1
34	Swears or curses at the child.	EA	CTSPC-PsA/NPM 06.1
35	Does not allow the child to take the proper medicine when the child had a diagnosed physical problem.	PN	NPM 08.0
36	Does not pay attention to the safety of the child.	PN	NPM 12.5
37	Allows the child to play in an unsafe environment.	PN	NPM 12.5
38	Leaves the child unsupervised.	PN	NPM 11.0
39	Does not take care of the child.	PN	CTQ-PN/NPM all
40	Leaves the child home alone, even though the child needs supervision.	PN	CTSPC-N/NPM 11.0
41	Hits the child badly enough to be noticed by others.	PA	CTQ-PA/NPM 04.2/0.3
42	Hits the child on some other part of the body besides the bottom with a hard object (e.g. belt, hairbrush, stick).	PA	CTSPC-SPA/CTQ-PA/NPM 04.3
43	Grabs the child around the neck and chokes him/her.	PA	CTSPC-VSPA/NPM 04.4/0.6
44	Knocks the child down.	PA	CTSPC-SPA/NPM 04.1
45	Pinches the child.	PA	CTSPC-MPA/NPM 04.6
46	Gives the child mostly unhealthy foods.	filler	n.a.
47	Allows the child to meet with people who are under the influence of illicit drugs.	EN	NPM 16.1
48	Does not allow the child to interact with other children or to make friends.	EN	NPM 17.7
49	His/her expectations of the child are too high.	EN	NPM 17.5
50	Does not allow the child to get the treatment he/she needs for a diagnosed emotional or behavioral problem.	EN	NPM 17.1
51	Does not look out for the child.	EN	CTQ-EN/NPM 15.1
52	Ties the child down to control his/her behavior.	EA	NPM 05.1
53	Criticizes the child.	EA	NPM 06.1
54	Intimidates the child by threatening to destroy the child's possessions.	EA	NPM 07.1
55	Punishes the child.	EA	MBQ^j^/NPM 06.1/07.3
56	Tells the child he/she wishes the child was never born.	EA	CTQ-EA/NPM 06.1/07.3
57	Shouts, yells, or screams at the child.	EA	CTSPC-PsA/NPM 06.1
58	Does not allow the child to get the treatment he/she needs for a diagnosed physical problem.	PN	NPM 08.0
59	Does not protect the child in potentially dangerous traffic situations.	PN	NPM 12.5
60	Is unable to offer the child a stable home.	PN	NPM 10.4
61	Refuses to offer the child the necessary physical care.	PN	NPM 10.1
62	Does not keep the child's clothes clean.	PN	CTQ-PN/NPM 12.3
63	Tries to hurt the child with a weapon.	PA	NPM 04.6
64	Physically abuses the child.	PA	CTQ-PA/NPM all
65	Hits the child on the bottom with a hard object (e.g. belt, hairbrush, stick).	PA	CTSPC-SPA/CTQ-PA/NPM 04.3
66	Beats the child up (i.e. hits child over and over again as hard as he/she can).	PA	CTSPC-VSPA/NPM 04.6
67	Throws the child (not as a game).	PA	CTSPC-SPA/NPM 04.1
68	Slaps the child on the hand, arm, or leg.	PA	CTSPC-MPA/NPM 04.2
69	Does not offer routine to the child.	EN	NPM 17.4
70	Fights with another family member in front of the child.	EN	NPM 15.2
71	Fails to be a good role model for the child.	EN	NPM 17.6
72	Is extremely overprotective of the child.	EN	NPM 17.3
73	Is not a source of strength for the child.	EN	CTQ-EN/NPM 15.1
74	Does not make the child feel loved.	EN	CTQ-EN/NPM 15.1
75	Threatens to initiate sexually inappropriate behavior towards the child.	EA	NPM 06.2
76	Ridicules the child.	EA	NPM 06.1
77	Teases the child.	EA	NPM 06.1/07.3
78	Makes the child feel hated by him/her.	EA	CTQ-EA/NPM 06.1
79	Says he/she will send the child away or kick the child out of the house.	EA	CTSPC-PsA/NPM 06.3
80	Leaves the child unattended for too long, considering the child's age.	PN	NPM 11.0
81	Is unable to provide warm clothes to the child when needed.	PN	NPM 12.3
82	Does not keep the child clean.	PN	NPM 12.2
83	Refuses to take care of the child.	PN	NPM 10.2
84	Is so drunk or high that he/she cannot take care of the child.	PN	CTSPC-N/CTQ-PN/NPM 19.3
85	Is not able to make sure the child gets the food he/she needs.	PN	CTSPC-N/CTQ-PN/NPM 12.1
86	Physically pushes the child.	PA	NPM 04.4
87	Hits the child so hard that the child needs to see a doctor.	PA	CTQ-PA/NPM 04.2/0.3
88	Burns or scalds the child on purpose.	PA	CTSPC-VSPA/NPM 04.6
89	Hits the child with a fist.	PA	CTSPC-SPA/NPM 04.5
90	Shakes the child.	PA	CTSPC-MPA/NPM 04.1

*Note:*

a *Type* refers to type of maltreatment: (PA) = physical abuse; (PN) = physical neglect; (EA) = emotional abuse; (EN) = emotional neglect

b NPM = Tweede Nationale Prevalentiestudie Mishandeling van Kinderen en Jeugdigen [11]

c CTQ = Childhood Trauma Questionnaire [12]

d CTSPC= Parent-Child Conflict Tactics Scale [13]

e PsA= Psychological aggression

f N= Neglect

g VSPA = Very severe physical assault (severe physical maltreatment)

h SPA: Severe physical assault (physical maltreatment)

i MPA = Minor physical assault (corporal punishment)

j MBQ: Maternal Behavior Q-sort [14].

**Table 2:** Percentage of Mothers Labeling MQS items as Maltreatment per Country (High to Low by Grand Mean Percentage).

Item #	Item content	Type^a^	Total	Range^b^	China	Iran	Netherlands	Portugal	S-Africa
43	Grabs the child around the neck and chokes child	(PA)	95	18	100	98	100	97	82
18	Uses a weapon to hit the child	(PA)	94	18	96	98	100	95	82
20	Threatens the child with a knife or gun	(PA)	94	14	86	98	100	97	90
75	Threatens inappropriate sexual behavior	(EA)	94	14	86	93	100	97	90
88	Burns or scalds the child on purpose	(PA)	94	20	96	98	100	97	80
63	Tries to hurt the child with a weapon	(PA)	92	12	86	98	99	95	80
64	Physically abuses the child	(PA)	92	11	92	89	97	97	86
66	Beats the child up	(PA)	91	20	84	93	100	93	80
87	Hits child so hard that it needs a doctor	(PA)	91	22	84	96	100	93	78
32	Threatens to kill the child	(EA)	91	15	84	91	99	95	84
19	Hits the child so hard that it leaves bruises	(PA)	88	17	78	93	95	90	80
41	Hits child noticeable by others	(PA)	85	18	72	89	100	86	74
42	Hits child with hard object (not on bottom)	(PA)	85	31	68	93	99	91	71
21	Kicks the child hard	(PA)	83	25	70	93	92	91	67
67	Throws the child	(PA)	80	30	72	89	88	79	69
58	Does not allow treatment for physical problem	(PN)	79	20	68	76	83	88	78
65	Hits child on bottom with hard object	(PA)	79	49	46	87	95	86	74
26	Uses illegal drugs in presence of child	(EN)	80	31	60	76	91	86	82
44	Knocks the child down	(PA)	80	36	64	78	100	79	74
89	Hits child with a fist	(PA)	79	38	54	87	92	84	76
22	Slaps the child on the face or head or ears	(PA)	79	33	56	89	89	77	80
52	Ties the child down to control it	(EA)	78	24	64	80	88	88	65
84	So drunk or high, incapable of care	(PN)	77	51	26	80	97	95	82
11	Emotionally abuses the child	(EA)	77	38	66	51	89	88	84
47	Allows child to meet with people on drugs	(EN)	75	14	66	78	80	75	74
35	Does not allow medicine when needed	(PN)	75	44	40	84	83	84	80
30	Locks child in closet as punishment	(EA)	74	35	68	51	86	81	80
56	Tells the child (s)he wished it was never born	(EA)	71	40	46	78	77	67	86
59	Does not protect child from dangerous traffic	(PN)	70	24	64	69	57	81	80
17	Does not provide doctor when needed	(PN)	70	43	42	64	85	83	69
16	Refuses to offer child shelter	(PN)	69	61	44	38	99	83	67
50	Does not allow care for emotional problems	(EN)	69	25	58	58	74	83	69
1	Unable to offer child safe home	(PN)	65	36	46	47	82	63	74
80	Leaves child unattended for too long	(PN)	65	32	46	78	60	74	69
61	Refuses the child necessary physical care	(PN)	64	40	42	47	82	75	65
40	Leaves child home alone	(PN)	64	44	42	51	39	75	86
83	Refuses to take care of the child	(PN)	63	56	38	44	94	60	67
78	Makes child feel hated by him/her	(EA)	62	36	44	53	80	61	65
15	Does not provide child with safe environment	(PN)	61	30	42	56	72	61	71
36	Does not pay attention to safety of the child	(PN)	61	29	40	58	65	68	69
14	No adequate care when child is ill	(PN)	60	78	8	58	74	86	67
3	Allows child to meet with drunk people	(EN)	58	44	22	76	62	60	74
8	Verbally aggressive to child	(EA)	57	32	36	51	68	63	63
38	Leaves child unsupervised	(PN)	57	33	44	51	60	61	67
85	Unable to make sure child gets food it needs	(PN)	56	45	36	29	68	74	67
31	Humiliates child in front of others	(EA)	56	13	48	53	60	56	61
79	Says he/she will kick child out of the house	(EA)	56	33	38	51	52	67	71
39	Does not take care of the child	(PN)	55	53	22	42	75	63	65
37	Allows child to play in unsafe environment	(PN)	53	23	38	51	55	60	61
27	Fails to find treatment for emotional problems	(EN)	52	47	22	58	51	61	69
34	Swears or curses at the child	(EA)	52	30	46	44	45	51	74
33	Says hurtful things to the child	(EA)	50	43	24	67	52	42	67
68	Slaps child on hand, arm, leg	(PA)	49	73	14	87	60	33	53
86	Physically pushes the child	(PA)	46	51	12	62	40	56	63
74	Does not make the child feel loved	(EN)	45	39	22	40	51	47	61
10	Purposely destroys child's favorite toys	(EA)	45	38	16	53	48	54	51
90	Shakes the child	(PA)	45	48	10	58	66	37	49
60	Unable to offer child a stable home	(PN)	45	41	24	29	51	51	65
9	Belittles the child	(EA)	45	29	32	31	60	51	47
45	Pinches the child	(PA)	45	15	52	49	49	37	41
4	Does not react to the child's emotions	(EN)	42	47	16	36	52	58	63
81	Unable to provide warm clothes when needed	(PN)	42	45	16	33	46	53	61
12	Calls the child dumb or lazy	(EA)	42	47	16	42	51	37	63
54	Threatens to destroy child's possessions	(EA)	40	42	16	58	52	33	39
7	Does not make the child feel important	(EN)	40	51	16	33	43	39	67
25	Is emotionally unavailable to the child	(EN)	40	25	30	33	42	40	55
57	Shouts, yells, or screams at the child	(EA)	39	42	14	56	46	30	53
76	Ridicules the child	(EA)	39	33	18	31	46	47	51
48	Does not allow child to play with other kids	(EN)	38	31	16	36	43	47	47
70	Fights with another relative in front of child	(EN)	38	40	36	51	19	35	59
29	Unable to show child that (s)he loves the child	(EN)	37	59	8	40	32	40	67
24	Screams at other relative in presence of child	(EN)	36	45	18	53	22	32	63
13	Threatens to hit child but does not actually do it	(EA)	36	56	2	58	34	53	35
23	Spanks the child on the bottom with bare hand	(PA)	34	49	10	58	22	40	45
6	Does not intervene when the child is aggressive	(EN)	34	37	18	33	28	39	55
51	Does not look out for the child	(EN)	34	69	2	33	71	53	53
73	Is not a source of strength for the child	(EN)	31	51	2	29	22	53	51
82	Does not keep child clean	(PN)	31	53	2	27	26	44	55
28	Does not feel close to the child	(EN)	29	53	2	22	32	30	55
71	Fails to be good role model for the child	(EN)	27	33	12	33	22	28	45
5	Does not offer enough structure to the child	(EN)	26	45	4	29	8	46	49
77	Teases the child	(EA)	25	24	14	38	15	28	35
62	Does not keep child's clothes clean	(PN)	24	59	2	20	14	25	61
55	Punishes the child	(EA)	22	38	14	40	3	21	41
53	Criticizes the child	(EA)	21	55	2	20	8	25	57
72	Is extremely overprotective of the child	(EN)	20	73	12	24	8	81	57
69	Does not offer routine to the child	(EN)	20	39	2	22	6	32	41
49	Has too high expectations of the child	(EN)	17	35	4	27	5	18	39

*Note:* A light grey marking in column 1 denotes items with a low range of percentages – meaning high agreement - between countries (< 25%), a dark grey marking denotes items with a high range of percentages – meaning low agreement - between countries (> 50%), no marking indicates percentages between 25% and 50%.

a *Type* refers to type of maltreatment: (PA) = physical abuse; (PN) = physical neglect; (EA) = emotional abuse; (EN) = emotional neglect

b *Range* reflects the difference between the lowest and highest percentages across the countries.

## 2 Experimental design, materials, and methods

### 2.1 Data collection

Participants were recruited via personal contacts, social media, and snowball sampling in Chile, a big state company in China, personal networks in Greece, a school for extracurricular lessons, personal network, and snowball sampling in Iran, toddler playgroups and preschools in the Netherlands, preschools, health clinics, and snowball sampling in Portugal, lists of participants of previous research projects in South Africa, personal and professional networks, and snowball sampling in Turkey, and personal networks and though an NGO attending to socio-economically vulnerable women in Uruguay. All participants signed an informed consent form. Data were collected using a survey and the Maltreatment Q-sort. Mothers filled in a short questionnaire (online before the home visit or during the home visit) about socio-demographic family characteristics including educational level, income, age, and number of children. Educational level and annual gross family income were both measured on a 5-point scale ranging from (1) lowest education/income bracket to (5) highest education/income bracket. Exact scale points where constructed per country to be suitable for the local context (see Mesman et al. [8] for more specific information about these measurements). Participants’ maltreatment attitudes were assessed using a Q-set of 90 items, the Maltreatment Q-Sort (MQS). This Q-set was developed by the authors and includes 22 items reflecting physical abuse, 22 items reflecting emotional abuse, 22 items reflecting physical neglect, and 22 items reflecting emotional neglect. The items were taken from the definitions used in the Dutch Second National Incidence Study of Child Abuse and Neglect (NPM-2010; Alink et al. [11]), items of the Childhood Trauma Questionnaire [12], items of the Parent-Child Conflict Tactics Scale [13], and items of the Maternal Behavior Q-sort [14]. There were 2 filler items. The MQS was piloted among ten developmental researchers from very different cultural backgrounds (China, Chile, Belgium, Egypt, Zambia, Canada, the UK, the Netherlands, and Vietnam) to ascertain the cross-cultural clarity of the instrument, as well as get a first sense of whether the instrument had the potential to yield individual differences in the rank ordering of the items. Both were confirmed, so that the instrument was then finalized without further changes.

The participants were first asked to sort the cards into three stacks from “least damaging to children” to “most damaging to children”. The participants were explicitly told that there are no correct or wrong answers and that it is all about their opinion regarding how damaging certain parenting behaviors are to child development. Any question they had concerning the meaning of an item was answered according to standardized item explanations in the protocol. After the participants distributed the cards across the three stacks, they were asked to sort each stack into three smaller stacks. After the participants distributed all cards across nine stacks, they were asked to evenly distribute the cards across the stacks until each stack consisted of 10 cards. To provide an additional visual aid to the scale of 9 stacks, the color of the anchor cards 1 to 9 were colored bright yellow (1 = least damaging), via darkening shades of orange (2–8) to bright red (9 = most damaging). Usually, a Q-sort instrument also includes a criterion sort that provides the ‘gold standard’ (usually devised by a small team of experts) to which participants’ sorts can be compared. However, the MQS does not have such a gold standard, because there is no single universally agreed-upon rank ordering of specific maltreating behaviors in terms of their potentially damaging effects on children.

In 5 out of 9 countries (China, Iran, Netherlands, Portugal, South Africa), additional information was obtained. After participants had completed the sorting task, they were asked to indicate from which stack onwards they thought (1) someone should intervene – without reference to who that would be; (2) they themselves would intervene; (3) a professional should intervene; (4) that the behaviors described on the cards should be considered child maltreatment. Thus, a participant might indicate for example that they thought all behaviors from the 4th stack onwards constitute maltreatment, putting all of the items in stacks 4 to 9 in the maltreatment category (reflecting 6 × 10 = 60 behaviors labeled as maltreatment). These indicated stacks thus represent thresholds for intervention and for the definition of maltreatment. The higher the threshold, the lower the number of behaviors seen as requiring intervention or as reflecting child maltreatment.

### 2.2 Preparing data for analyses

**Fig. 1:**
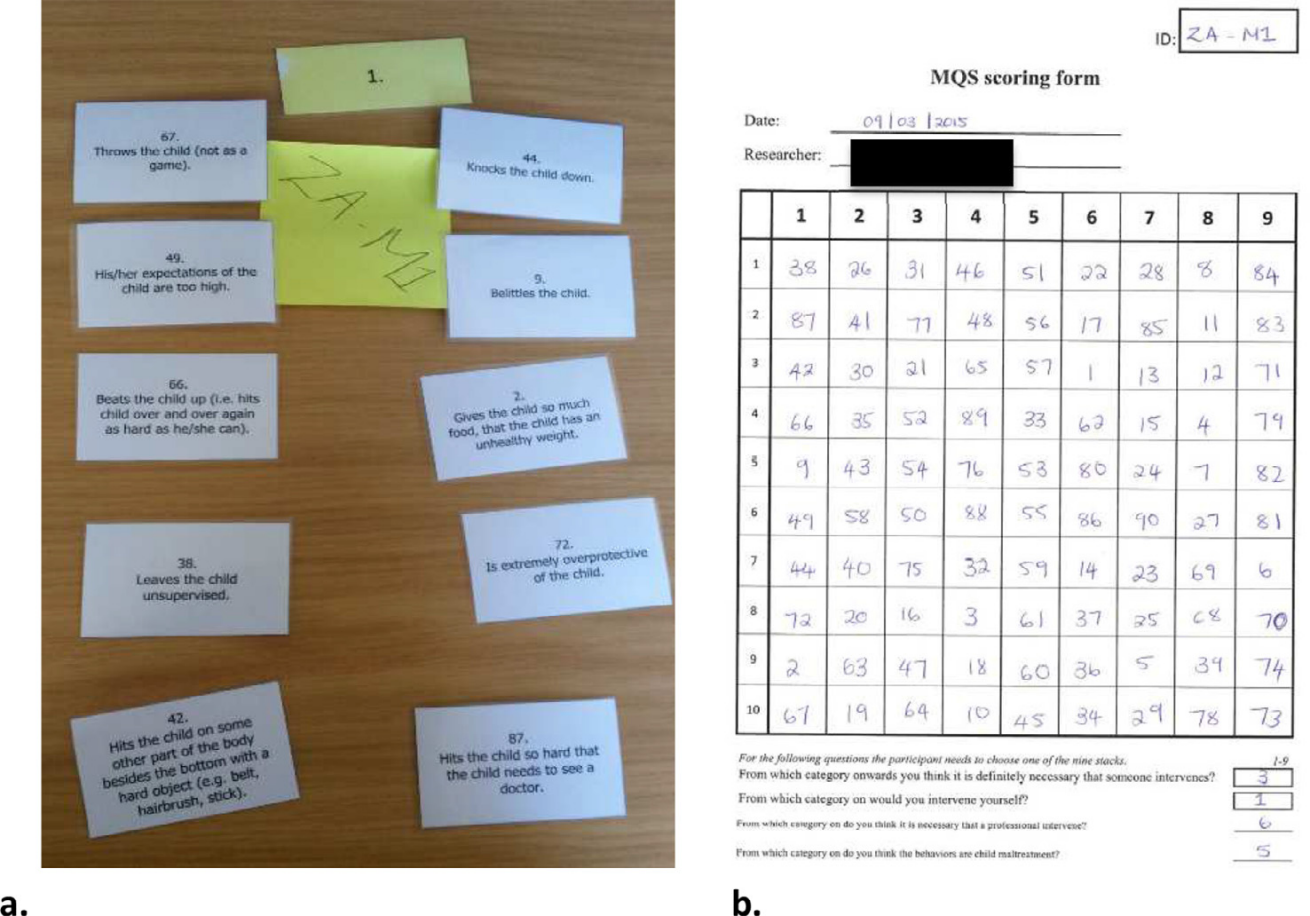
Example of a picture of the 10 cards, stack number, and ID-number of one stack (a) and an example of a MQS scoring form (b).

To analyze the data IBM SPSS statistics is used. It is important that data-entry is done in the correct way to be able to analyze Q-sort data. To record how each participant sorted the 90 cards, pictures are taken of the nine stacks including the ID-number, the 10 cards belonging to the stack, and the stack number, after administering the MQS (see Fig. 1a for an example). To avoid taking up too much time of the participants, collect the 9 stacks in 9 separate envelopes (one envelope per stack with the 10 cards and the stack number) and make the pictures at a later time point. Use the pictures to fill in the data on a scoring form (see Fig. 1b). The order of the 10 cards within each stack is not relevant, as long as the 10 item numbers are filled in below the correct stack number. The scoring form could be used to enter the data in SPSS.

The dataset ‘Qsort-Datafile’ [1] is an example of how to correctly enter Q-sort data in SPSS. Each column represents a stack (from 1 to 9) and the rows represent the 10 cards placed on each stack. The Q-sort data of all participants can be entered in the same file below each other, but it is important to leave one blank row in between the data of different participants. Before analysis, the data need to be restructured to make sure that columns represent participants and that rows represent the MQS cards. To do this, two syntaxes developed by Van Ginkel [2,3] are needed. Both syntaxes should be saved in the same location. Only the syntax file ‘RunReshape Qsorts’ needs to be opened and edited. There are six rows in the syntax file;

• Row 1: type the correct location where the syntax file ‘SyntaxReshape’ is saved.
• Row 2: type the location of the data file with all raw Q-sort data (in this example the file is called ‘Qsort-Datafile.sav’).
• Row 3: type the location where the new file will be saved as well as the name of the new file (for example ‘Qsort-NewDatafile.sav’). Make sure the name of the new file is different from the file with the raw Q-sort data.
• Row 4: type the names of the new variables. Each variable in the new dataset represents the Q-sort data of one participant. In this example the variables are called ‘Q-sort’ (participant 1 will become Qsort1, participant 2 Qsort2 and so on), but this could be changed to any desired variable name.
• Row 5 does not have to be edited.
• Row 6: type the correct number of participants (i.e., the number of Q-sorts entered in the ‘Qsort-Datafile’ SPSS file).

Make sure only the syntax ‘RunReshape Qsorts’ is opened (the syntax ‘SyntaxReshape’ and the data file with all Q-sort data, in this example ‘Qsort-Datafile’, need to be closed). Run the syntax. A new data file is made. Data file ‘Qsort-NewDatafile’ [4] is an example of how the new data file should look like. If an error occurs while running the syntax check whether there are spaces in the location names in the syntax (these should be deleted) and whether the Q-sort data are filled in correctly (all 90 items should be entered and there should be no double entries of the same card number). The new data file can be used for analyses. Data set ‘MQS Output all mothers’ [9] is the data file with the restructured data of the Q-sorts of 466 mothers from nine different countries of the study of Mesman et al. [8].

### 2.3 Data analyses

One way to analyze the data in the new file (‘Qsort-NewDatafile’) is to calculate the agreement of mothers within and between countries on how they constructed the Q-sorts. This is done by calculating correlations between the Q-sort of all mothers from one country and between the Q-sort of each mother from one country and each mother of another country. To do this two syntaxes can be used, also developed by Van Ginkel [5,6]. Similar as before, only the syntax ‘RunAutomatedRestructuring’ needs to be opened and adapted. There are again six rows;

• Row 1: type the location of the syntax file (‘SyntaxAutomatedRestructuring’) needed to run the current syntax.
• Row 2: type the location of the data file that needs to be used for analyses (e.g., ‘Qsort-NewDatafile’).
• Row 3: type the location and name of the new data file (e.g., ‘Qsort-NewDatafile-mothersCLmothersNL.sav’).
• Row 4: type the variable labels of the two groups used to calculate the agreement. In the example the first group consists of Chilean mothers and the second group of Dutch mothers. Therefore the variable labels ‘MothersCL’ and ‘MothersNL’ are used. This can however be changed to any desired variable names.
• Row 5: type the first variable numbers of the two groups. Each participant equals one variable (i.e., column). In the current example there are eight mothers in total, five Chilean mothers and three Dutch mothers. The Chilean mothers start at variable 1 and the Dutch mothers at variable 6. Therefore type 1, 6 in row 5.
• Row 6: type the end variable numbers. In the current example the Chilean mothers end at variable 5 and the Dutch mothers at variable 8, so type 5, 8 in the last row.

When all six row are edited run the syntax. Again make sure both the data file ‘Qsort-NewDatafile’ and the other syntax file ‘SyntaxAutomatedRestructuring’ are closed and only the syntax file ‘RunAutomatedRestructuring’ is open. Dataset ‘Qsort-NewDatafile-mothersCLmothersNL.sav’ [7] is an example of how the new data file should look like. There are three variables in the new data file; ‘MQS 11′ which are the correlations of the Q-sorts between the mothers of the first group; the Chilean mothers. ‘MQS12’ represents the correlations of the Q-sorts between the mothers of the two groups, in this case between the Chilean and Dutch mothers. Finally variable ‘MQS22’ represents the correlations of the Q-sorts between the mothers of group two, the Dutch mothers in the current example. The syntax ‘SyntaxAutomatedRestructuring’ creates the variable names (e.g., MQS11). The variable labels show which variable represents the correlations between which group(s), therefore it is important to use the correct variable labels in Row 4. The variables with the agreement of the Q-sorts within and between groups can be compared by calculating ranges, means, standard deviations and 95% confidence intervals.

Another way to analyze the Q-sort data is by calculating the mean stack on which mothers placed the items reflecting the four subscales of child maltreatment. To do this the ‘participants-as-variables’ data file should first be restructured to a ‘items-as-variables’ data file. This can be done by transposing the data so that variables becoming rows and one row now represents one participant (instead of one column representing one participant). When the data are transposed, background variables can be added as well as other variables, including the data about threshold for intervention and threshold for defining child maltreatment. With this data file the four subscales can be created by calculating the mean of the items reflecting the different subscales (see Table 1). The averages can be compared within and between countries. Data file ‘MQS Datafile’ [10] is an example of what the data look like.

## Appendix Supplementary materials

Image, application 1
